# Differences in inflammation biomarkers between patients with paroxysmal and persistent atrial fibrillation in the femoral vein and coronary sinus blood samples; a cohort study

**DOI:** 10.1093/ehjopen/oeaf089

**Published:** 2025-07-15

**Authors:** Carlos Valera Soria, Carl-Johan Carlhäll, Lars O Karlsson, Johan Lindbäck, Ziad Hijazi, Emmanouil Charitakis

**Affiliations:** Department of Cardiology, Linköping University Hospital, Kardiologiska kliniken, Universitetssjukhuset, Linköping 581 85, Sweden; Department of Health, Medicine and Caring Sciences, Linköping University, SE-58183 Linköping, Sweden; Department of Health, Medicine and Caring Sciences, Linköping University, SE-58183 Linköping, Sweden; Department of Clinical Physiology, Linköping University Hospital, Fysiologiska kliniken, Universitetssjukhuset, 581 85 Linköping, Sweden; Department of Cardiology, Linköping University Hospital, Kardiologiska kliniken, Universitetssjukhuset, Linköping 581 85, Sweden; Department of Health, Medicine and Caring Sciences, Linköping University, SE-58183 Linköping, Sweden; Uppsala Clinical Research Center, Uppsala University, Uppsala Science Park, HubbenDag Hammarskjölds väg 38, SE-751 85 Uppsala, Sweden; Uppsala Clinical Research Center, Uppsala University, Uppsala Science Park, HubbenDag Hammarskjölds väg 38, SE-751 85 Uppsala, Sweden; Department of Medical Sciences, Uppsala University, Akademiska sjukhuset, ingång 40, plan 5, 751 85 Uppsala, Sweden; Department of Health, Medicine and Caring Sciences, Linköping University, SE-58183 Linköping, Sweden; Karolinska Institutet, Department of Medicine, Solna Karolinska University Hospital, D1: 04171 76 Stockholm, Sweden

**Keywords:** Atrial fibrillation, Coronary sinus, Inflammation, Biomarkers, Pathophysiological features

## Abstract

**Aims:**

The association between inflammation and atrial fibrillation (AF) is evident, but assessing the specific inflammatory pathways involved in the pathogenesis remains complex. This study aimed to identify inflammatory biomarkers associated with paroxysmal (PAF) and persistent (PeAF) AF by evaluating blood samples from the intra- and extracardiac space.

**Methods and results:**

This is an observational, cross-sectional, single-centre study. A total of 92 inflammatory biomarkers were analyzed from blood samples taken from the coronary sinus (CS) and the femoral vein (FV) in 88 patients with AF who had been referred for catheter ablation at the Linköping University Hospital, Sweden. The concentrations of the biomarkers were compared between PAF and PeAF patients in the CS and FV. Significant differences in concentration were found in 36 of 92 biomarkers. Among these, 12 proteins stand out for exhibiting a higher concentration in PeAF patients: Interleukin 6 (IL-6), CUB domain-containing protein 1 (CDCP1), Interleukin 18 receptor 1 (IL-18R1) and cystatin D (CST5) in the FV, β nerve growth factor (β-NGF) and tissue growth factor α (TGF-α) at the CS level, as well as interleukin 18 (IL-18), chemokine ligand 3 (CCL-3) and tumour necrosis factor superfamily 14 (TNFSF-14) in both FV and CS. Moreover, chemokine ligand 25 (CCL-25), chemokine ligand 28 (CCL-28), and artemin (ARTN) were found at a higher concentration in the CS in the overall population.

**Conclusion:**

This study supports the involvement of TNFSF-14, IL-6, and IL-18 in the pathogenesis and maintenance of PeAF. Furthermore, it identifies β-NGF and TGF-α as potential participants in the pathogenesis and/or maintenance of PeAF locally in the atria. Novel inflammatory biomarkers, mainly chemokines, are also identified as possibly involved in the pathophysiology of AF.

## Graphical Abstract

**Graphical Abstract:**
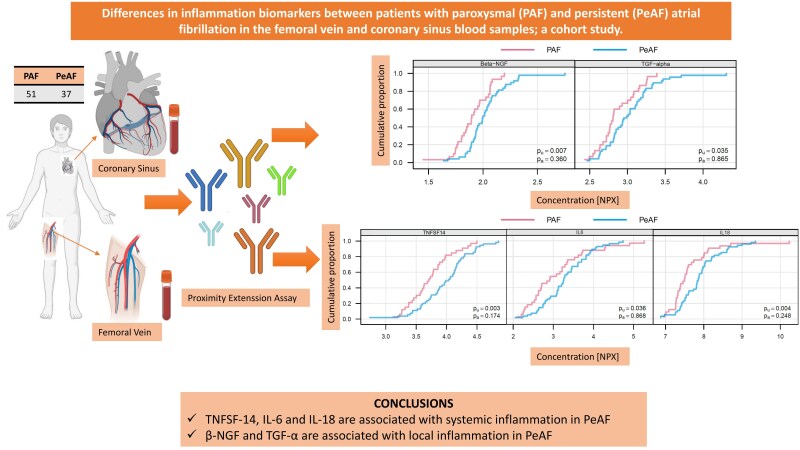

What is already known in this topic

- Different inflammatory mediators have been addressed, and some others have been hypothesized, to play a role in the pathogenesis of AF.^1^ It has been suggested that cardiomyocytes, as well as immune cells, can release inflammatory mediators. Cardiac fibroblasts become activated by cytokines, growth factors and adipokines often associated with metabolic disorders and inflammatory diseases that commonly coexist with AF. As a result, inflammation can be an underlying factor in the development of atrial fibrosis, promoting AF progression.^2^

What this study adds

- The findings obtained after analyzing this wide range of inflammatory biomarkers not only support some of the previous evidence in the field but also identify new pathways that may be involved in the development and progression of AF, representing a step forward in the research-oriented towards both the prevention of AF and the progression from PAF to PeAF.

How this study might affect research, practice or policy

- Ideally, these findings need to be validated and could eventually lead to the utilisation of some of these proteins for predictive or prognostic use to potentially assess the probability of AF progression from PAF to PeAF. Such a concept may also provide decision support regarding rhythm control strategies in patients with AF based on the number of risk factors, elevated inflammatory biomarkers, and low or moderate grade of symptoms.

## Introduction

Atrial fibrillation (AF) is the most common sustained cardiac arrhythmia,^3^ with a prevalence of 6% in people over 65 years old and 10% in people over 80.^4^ AF can be clinically categorized into two main groups, paroxysmal and non-paroxysmal, based on the duration and intervention required to terminate the arrhythmia.^5^ Paroxysmal AF (PAF) refers to instances where the arrhythmia self-terminates within a period of fewer than 7 days. Within the non-paroxysmal group, persistent AF (PeAF) is characterized by self-termination beyond a 7-day timeframe or that necessitates cardioversion to restore sinus rhythm.^6^

A complete understanding of the pathophysiology underlying AF remains to be elucidated. Still, inflammation is closely associated with the development and maintenance of AF by its implication in processes such as oxidative stress, cell death, and atrial fibrosis, which contribute to the progression of atrial remodelling and, thereby, AF burden.^2^ It has been suggested that cardiomyocytes, as well as immune cells, can release inflammatory mediators. Cardiac fibroblasts become activated by cytokines, growth factors and adipokines often associated with metabolic disorders and inflammatory diseases that commonly coexist with AF. As a result, inflammation can be an underlying factor in the development of atrial fibrosis, promoting AF progression.^2^

The use of a custom-made proteomics chip based on proximity extension assay technology (PEA) provides the possibility to simultaneously analyze a high number of inflammatory proteins that are thought to be associated with cardiovascular disease. The essentials of this method are that each protein is assessed using a pair of specific antibodies that bind to complementary oligonucleotides on the protein, granting the possibility to measure the protein concentration using quantitative real-time polymerase chain reaction (PCR).^7^ PEA assays have shown high reproducibility and repeatability.^8^

Different inflammatory mediators have been addressed, and some others have been hypothesized, to play a role in the pathogenesis of AF.^1^ However, the involvement of different inflammatory pathways in PeAF, in contrast to PAF, has not yet been clarified. Moreover, there is limited knowledge about the local production, tissue infiltration, and activation of inflammatory biomarkers that occur in the pathophysiology of different AF types, which requires further assessment.

In the present study, we aimed to single out inflammatory biomarkers to identify the inflammatory pathways that may be associated with PeAF intra- and extracardially, by comparing blood samples obtained from the coronary sinus (CS) and the femoral vein (FV) in patients planned for pulmonary vein isolation.

## Method

### Study design and measurement

This was an observational, cross-sectional, single-centre cohort obtained from the SMURF (Symptom burden, Metabolic profile, Ultrasound findings, Rhythm, neurohormonal activation, haemodynamics, and health-related quality of life in patients with AF) study carried out between January 2012 and April 2014.^9^ Patients referred to the University Hospital in Linköping, Sweden, for radiofrequency ablation (RFA) due to AF were screened for potential participation.

The enrolment protocol of patients is described in detail in the protocol article,^9^ and summarized below.

#### Screening before admission and ethical considerations

The protocol for this study was approved by The Regional Ethical Review Board of the Faculty of Health Sciences, Linköping, Sweden. Trial registration number NCT01553045. Oral and written consent was provided by all the patients, and the study adhered to the principles outlined in the Declaration of Helsinki.^10^ The inclusion and exclusion criteria are described in *Table 1*.

**Table 1:** Inclusion and exclusion criteria

Inclusion criteria	Exclusion criteria
1. Age 18 years or older with paroxysmal or persistent AF.	1. Patients with previous catheter or surgical AF ablation.
2. Patients referred for RFA.	2. Patients with previous or expected heart surgery.
3. Patients with sufficient knowledge of the Swedish language to independently fill out the study questionnaires.	3. Patients with severe heart failure with LVEF < 35%.
	4. Patients with acute coronary syndrome during the past 3 months.

AF, atrial fibrillation; LVEF, left ventricular ejection fraction; RFA, radiofrequency ablation.

#### Inclusion day

Prior to the RFA, a comprehensive baseline assessment was conducted, which included previous medical history, physical examination, and a 12-lead electrocardiogram. In addition, all patients underwent a transthoracic echocardiogram, transoesophageal echocardiogram, and a heart computerized tomography scan per standard clinical protocol.

#### Ablation day

Before the RFA procedure, patients were catheterized, and blood samples were collected both peripherally (from the FV) and centrally (from the CS).^1^ The primary goal of the procedure was to electrically isolate all pulmonary veins through antral ablation. The ablation protocol has been described previously.^9^

### Biochemical analyses

Blood samples were obtained in plastic EDTA vials. Subsequently, the vials were centrifuged at 3100 g for 20 min. Plasma was then separated and preserved by freezing at −70°C for long-term storage. None of the samples were thawed more than two times.

In our proteomics analysis, the Proseek Multiplex PEA Inflammation panel provided by Olink Proteomics in Uppsala, Sweden, was used.

The 92 inflammatory biomarkers included in this panel were pre-selected by Olink Proteomics based on prior research, published literature, and clinical relevance to inflammatory and cardiovascular disease. This selection aligns with the aims of this study. The Olink Target 96 Inflammation panel has demonstrated utility in previous studies investigating similar pathophysiological mechanisms.^7,11^

The biochemical analysis was conducted at the Clinical Biomarkers Facility at the Science for Life Laboratory at Uppsala University, Uppsala, Sweden. This panel allowed us to simultaneously measure 92 different biomarkers by pairing single-strand oligonucleotide-labeled antibodies with target proteins from plasma samples. Subsequently, the formation of double-stranded DNA amplicons facilitated quantification using the Fluidigm BioMarkTM HD real-time PCR platform. The values are reported in terms of Normalized Protein Expression (NPX) and have been log2-transformed.

### Statistical methods

For the analysis of the baseline characteristics, continuous variables are summarized with means and standard deviations (SD). Non-normally distributed variables are summarized with medians and interquartile range (IQR), and categorical data with counts and percentages in brackets.

The distribution of each PEA marker is presented using empirical cumulative distribution function (ECDF) plots and compared between groups using the Wilcoxon-Mann–Whitney test. Multiplicity adjustments were done by controlling the false discovery rate and both unadjusted and adjusted *P*-values are presented in the ECDF plots. Measurements from FV and CS within the same patient were compared using the Wilcoxon signed ranks test. The ratio between the measurements was plotted using ECDF plots and compared between locations and between AF types using Wilcoxon-Mann–Whitney tests.

Reported *P* values are two-sided; a *P*-value < 0.05 was considered statistically significant. The analyses were performed using SPSS 27.0 (SPSS, Chicago, IL, USA).

## Results

Out of the 88 randomly selected participants, 51 had a diagnosis of PAF and 37 PeAF, respectively. The baseline characteristics are presented in *Table 2*. In the studied cohort, no patient was known to suffer from previous myocarditis or severe kidney disease (GFR <30 mL/min/1.73 m²). However, 25% of the patients had impaired renal function, which, although proportionally similar in both groups, could influence the interpretation of the obtained results. Four patients with inflammatory or autoimmune disease were include, given the low prevalence and the proportionally similar distribution in both groups, these comorbidities are unlikely to have influenced the overall results. Clinically relevant comorbidities such as heart failure and hypertension were more frequent in PeAF patients in contrast to PAF patients, including associated medications such as renin-angiotensin-aldosterone system blockers. A higher proportion of patients in the PAF group were on antiarrhythmic medication. The proportion of patients presenting with AF on arrival to the ablation lab was significantly higher in the PeAF group. The levels of natriuretic peptides were higher in PeAF patients compared with PAF patients. Patients with PeAF presented a larger left atrial volume than those with PAF.

**Table 2:** Baseline characteristics

	Total (*n* = 88)	Paroxysmal AF (*N* = 51)	Persistent AF (*N* = 37)
Age, yrs (SD)	60.5 (± 10, 6)	60 (±11, 6)	61.5 (±9, 2)
Male, *n* (%)	62 (70%)	34 (66, 7%)	28 (75, 7%)
Female, *n* (%)	26 (29%)	17 (33, 3%)	9 (24, 3)
BMI (kg/m^2^) (SD)	27.9 (±4, 3)	27.2 (±3, 8)	29.1 (± 4, 8)
Hypertension	39 (44, 3%)	18 (35, 3%)	21 (56, 8%)
Diabetes Mellitus	7 (8%)	3 (5, 9%)	4 (10, 4%)
Heart Failure	8 (9, 1%)	0 (0%)	8 (21, 6%)
Structural Heart Disease	9 (10, 2%)	6 (11, 8%)	3 (8, 1%)
Ischaemic Cardiomyopathy	6 (6, 8%)	6 (11, 8%)	0 (0%)
Dilaterad Cardiomyopathy	2 (2, 3%)	0	2 (5, 4%)
Hypertrophic Cardiomyopathy	1 (1, 1%)	0	1 (2, 7%)
CKD (GFR < 60 mL/min/1.73 m^2^)	22 (25%)	13 (25, 5%)	9 (24, 3%)
Stroke/TIA	7 (8%)	5 (9, 8%)	2 (5, 4%)
Cancer disease	2 (2, 3%)	1(1, 9%)	1(2, 7%)
Inflammatory and autoimmune disorders	4 (4, 5%)	3 (5, 9%)	1 (3, 7%)
Ulcerative Colitis	2 (2, 3%)	2 (3, 9%)	0 (0%)
Rheumatoid Artrit	1 (1, 1%)	1 (2%)	0 (0%)
Psoriasis	1 (1, 1%)	0 (0%)	1 (3, 7%)
CHA_2_DS_2_VASc (SD)	2.0 (± 1, 52)	1.5 (± 1, 48)	2.1 (±1, 54)
Beta-blocker	70 (79, 5%)	36 (70, 6%)	34 (91, 9%)
AAD	39 (44, 3%)	25 (49%)	14 (37, 8%)
Amiodarone	22 (25%)	9 (17, 6%)	13 (35, 1%)
Flecainide	13 (14, 8%)	12 (23, 5%)	1 (2, 7%)
Dronedarone	7 (8%)	6 (11, 8%)	0 (0%)
ACEi or ARB	39 (44%)	15 (29, 4%)	24 (64, 9%)
Statins	23 (26, 1%)	12 (23, 5%)	11 (29, 7%)
Cortisone	4 (4, 5%)	4 (7, 8%)	0
Methotrexate	1 (1, 1%)	1 (2%)	0
AF at the ablation lab	34 (38, 6%)	5 (9, 8%)	29 (78, 4%)
Complications^a^	5 (5, 7%)	1 (2%)	4 (10, 8%)
MR-proANP (pmol/L) (IQR)	164 (106–211)	128 (93–160)	215 (125–263)
NT-proBNP (pg/mL) (IQR)	458 (125–570)	223 (72–264)	789 (340–980)
LAVmax (mL) (CI 95%)	61 (57–64)	54 (49–60)	66 (59–74)
EF < 45%	15 (17%)	1 (2%)	14 (37, 8%)

Values are mean (± SD), mean (IQR), mean (CI 95%) or *n* (%). Baseline data are presented for all available patients (*N* = 88).

^a^Reported complications were cardiac tamponade, pericardial effusion, pseudoaneurysm, and larger than normal haematoma of the groin.

AAD, antiarrhythmic drugs; ACEi, angiotensin-converting enzyme inhibitor; AF, atrial fibrillation; ARB, angiotensin receptor blocker; BMI, body mass index; CKD, chronic kidney disease; EF, ejection fraction; GFR, glomerular filtration rate; LAVmax, maximum left atrial volume; MR-proANP, midregional pro-atrial natriuretic peptide; NT-proBNP, N-terminal pro b-type natriuretic peptide; TIA, transient ischaemic attack.

### Biomarkers concentration in the CS

**Figure 1:**
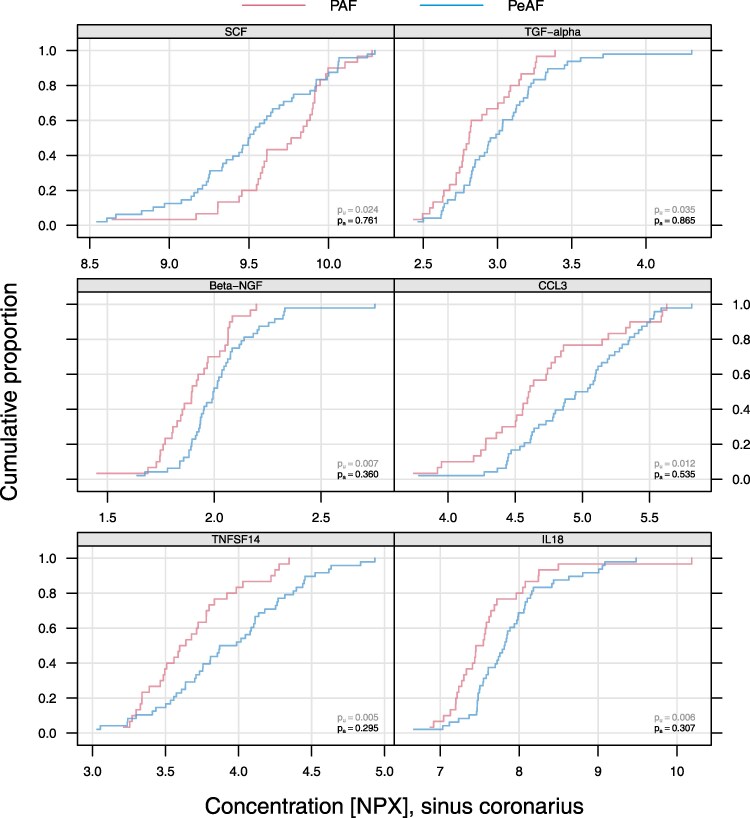
Biomarkers exhibiting a significant concentration difference in blood samples obtained from the CS in PAF and PeAF patients. The figure presents cumulative distribution curves for inflammatory markers (TNFSF14, IL-18, Beta-NGF, CCL3, SCF, and TGF-alpha) measured in the CS. The *x*-axis represents the concentration of each biomarker in normalized protein expression units, while the *y*-axis denotes the cumulative proportion of patients. The distribution is shown separately for patients with PAF and PeAF. The unadjusted *P*-value (*pu*) and adjusted *P*-value (*pa*) for group comparisons are reported for each biomarker.

When comparing the distributions of the concentration of the inflammatory biomarkers at the level of the CS, we identified that six of the 92 proteins showed a statistically significant difference (see Supplement  *S1*). Tumour necrosis factor superfamily fourteen (TNFSF-14), interleukin 18 (IL-18), beta-nerve growth factor (β-NGF), chemokine ligand 3 (CCL 3), and tissue growth factor alpha (TGF-α) were significantly higher in PeAF group, whereas the Skp Cullin F-box containing complex (SCF complex) was the only protein that showed a higher concentration in the PAF group (*Figure 1*).

### Biomarkers concentration at the FV

**Figure 2:**
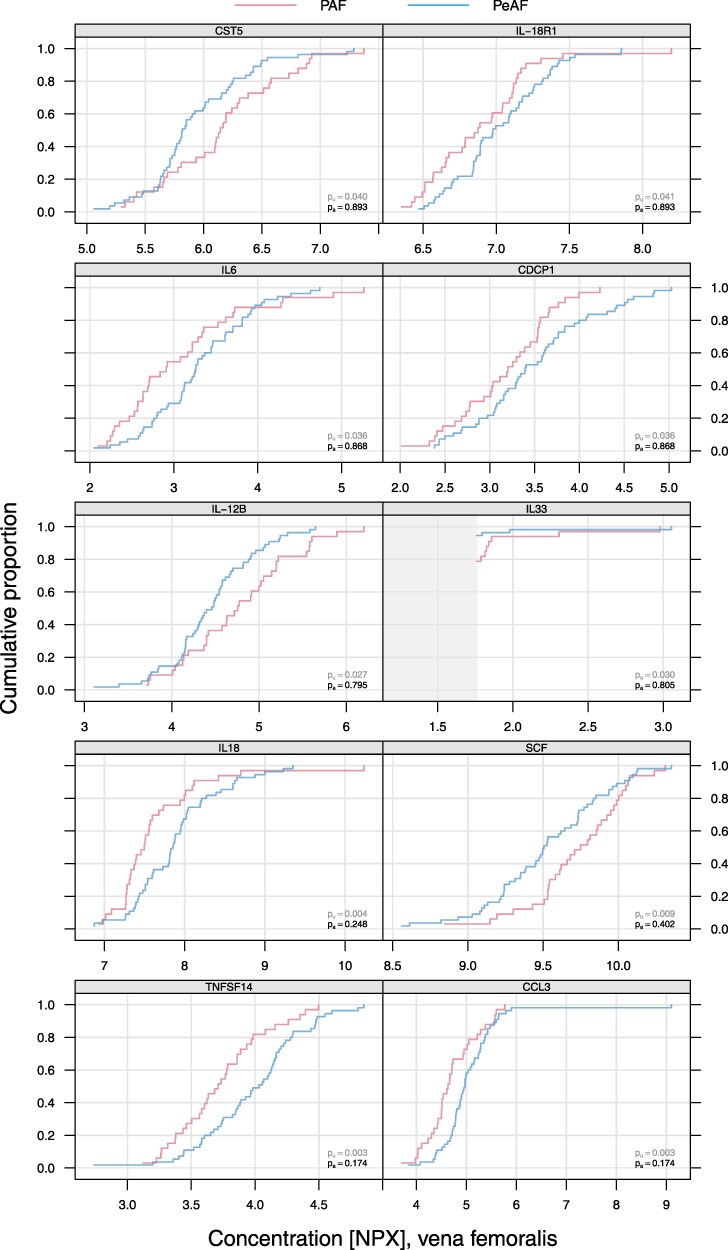
Biomarkers exhibiting a significant concentration difference in blood samples obtained from the FV in PAF and PeAF patients. The figure shows cumulative distribution curves for inflammatory markers (TNFSF14, CCL3, IL-18, SCF, IL-12B, IL-33, IL-6, CDCP1, CST5, and IL-18R1) measured in the FV. The *x*-axis represents the concentration of each biomarker in normalized protein expression units, while the *y*-axis denotes the cumulative proportion of patients. The distribution is presented separately for patients with PAF and PeAF. The unadjusted *P*-value (*pu*) and adjusted *P*-value (*pa*) for group comparisons are provided for each biomarker.

The peripheral blood samples analysis revealed 10 inflammatory biomarkers with a statistically significant concentration difference between the PAF and PeAF groups (see Supplement  *S2*). Among these proteins, TNFSF-14, CCL-3, CUB domain-containing protein (CDCP1), Interleukin 6 (IL-6), interleukin 18 receptor 1 (IL18R1), and IL-18 exhibited a higher concentration in the PeAF group. SCF complex, together with interleukin 12 B (IL-12B), interleukin 33 (IL-33), and Cystatin-D (CST5), showed a significantly higher concentration in the PAF group (*Figure 2*).

### Biomarkers concentration ratio between the CS and FV

**Figure 3:**
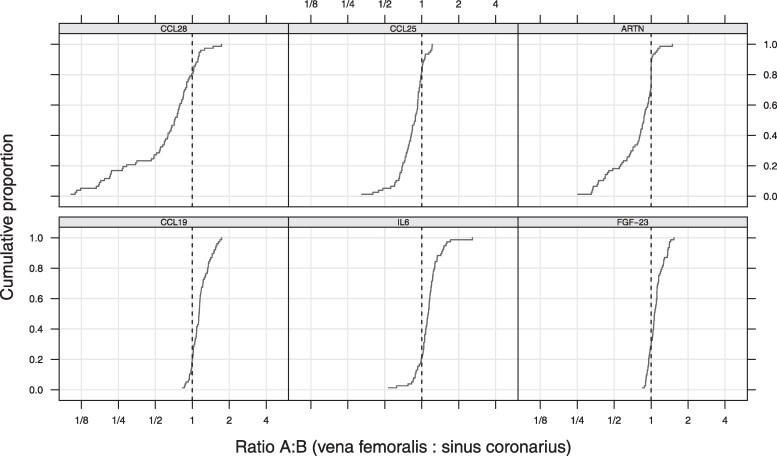
Biomarkers exhibiting a significant FV: CS concentration ratio difference in the overall population. The figure illustrates the cumulative distribution of the ratio of biomarker concentrations between the FV and CS for selected markers (CCL19, IL-6, FGF-23, CCL28, CCL25, and ARTN). The *x*-axis represents the ratio of concentrations (FV: CS) on a logarithmic scale, while the *y*-axis denotes the cumulative proportion of patients.

To compare the concentrations obtained in blood samples from the CS and FV in the total population, these were expressed and analyzed as concentration ratios between CS and FV, showing 27 proteins with a statistically significant concentration difference (see Supplement  *S3*). Among these, chemokine ligand twenty-eight (CCL-28), chemokine ligand twenty-five (CCL-25) and artemin (ARTN) were the proteins exhibiting the highest concentration in the blood samples taken from the CS compared with those obtained from the FV. Chemokine ligand nineteen (CCL-19), fibroblast growth factor 23 (FGF-23), and interleukin 6 (IL-6) were found to be present at a higher concentration in the FV compared with the CS (*Figure 3*). The main results are summarized in *Table 3*.

**Table 3:** Summary of the main findings

Inflammatory biomarker	PAF vs. PeAF concentration difference		CS vs. FV Concentration difference
	Coronary Sinus	Femoral Vein	
CCL-25	N	N	CS > FV
CCL-28	N	N	CS > FV
ARTN	N	N	CS > FV
Β-NGF	PeAF > PAF	N	N
TGF-α	PeAF > PAF	N	N
TNFSF-14	PeAF > PAF	PeAF > PAF	N
CCL-3	PeAF > PAF	PeAF > PAF	N
IL-18	PeAF > PAF	PeAF > PAF	N
IL-6	N	PeAF > PAF	CS < FV
IL-18R1	N	PeAF > PAF	N
SCF-complex	PeAF < PAF	PAF > PeAF	N
IL-12B	N	PeAF < PAF	N
CDCP1	N	PeAF > PAF	N
CST5	N	PeAF < PAF	N
CCL-19	N	N	CS < FV
FGF-23	N	N	CS < FV

N, No significant concentration difference.

## Discussion

Systemic inflammation is now recognized to represent a risk factor for cardiovascular disease and is also associated with the maintenance of AF.^12^ A local inflammatory process at the level of the atria has also been described in relation to AF. The infiltration of inflammatory cells into the atrial tissue, with particular emphasis on the infiltration of CD68 positive macrophages, as well as increased production of IL-6 and tissue growth factor β (TGFβ) by macrophages in patients with AF are some of the described mechanisms contributing to an increased expression of inflammatory cytokines such as IL-6, interleukin 8, interleukin 10 and TNF. Moreover, it has been suggested that inflammatory cell infiltration directly contributes to cytokine production at a local level.^13^

Interesting hypothesis have been raised regarding the contribution of AF to inflammation. A low-grade inflammatory response to cellular damage, associated to AF induced alterations in calcium homeostasis, leads to structural and electrical alterations in the atria.^14^ Harada *et al*.^15^ described how AF could contribute to the activation of rein-angiotensin-aldosterone system and promote inflammation.^15^ Previous studies have shown a decline in inflammatory proteins in the absence of AF after successful ablation as well as higher levels of inflammatory proteins in patients more prone to AF recurrence following cardioversion.^16^

In this biomarker study, we aimed to compare the concentration of 92 protein biomarkers reflecting different inflammatory pathways, measured by PEA multiplex and conventional immunoassays, in blood samples taken from the CS and FV in PAF and PeAF patients.

The PEA technique employs biomarker assays that yield values presented in relative units known as NPX values. While the PEA technique is highly effective for screening purposes, clinical applications may require absolute concentrations. Nevertheless, prior validation studies have shown that biomarkers analyzed using the PEA technique exhibit strong concordance with established immunoassays, making them suitable for further investigation^17^

The CS was chosen as the site for intracardiac blood sample measurement to enhance the sensitivity of biomarker detection,^18^ potentially providing a more comprehensive representation of cardiac inflammation. Although the left atrium, and more specifically the pulmonary veins, is the primary site of AF in the absence of structural heart disease,^19^ sampling from the CS may offer insights into broader myocardial inflammatory processes. However, since the CS drains blood from both the atria and ventricles, the measured inflammatory markers may not be exclusively reflective of atrial pathology.

In addition, we analyzed the differences in the concentrations of biomarkers identified in CS and FV samples from PAF and PeAF and compared both groups. Furthermore, we compared the biomarkers concentration in the CS and the FV in the total population.

CCL-25, CCL-28, and ARTN showed a higher presence in the CS, suggesting a local production in the heart. Β-NGF and TGF-α were identified at higher CS concentrations in PeAF patients. TNFSF-14, CCL-3, and IL-18 stood out by showing a stronger association with PeAF, with a significantly higher concentration at both CS and FV levels. Thus suggesting a systemic implication of IL-6, IL-18R1, and CDCP1 in relation to PeAF given by their higher concentration in FV samples compared with PAF patients.

### AF promotes inflammation and atrial remodelling

#### Interleukin 6

**Figure 4:**
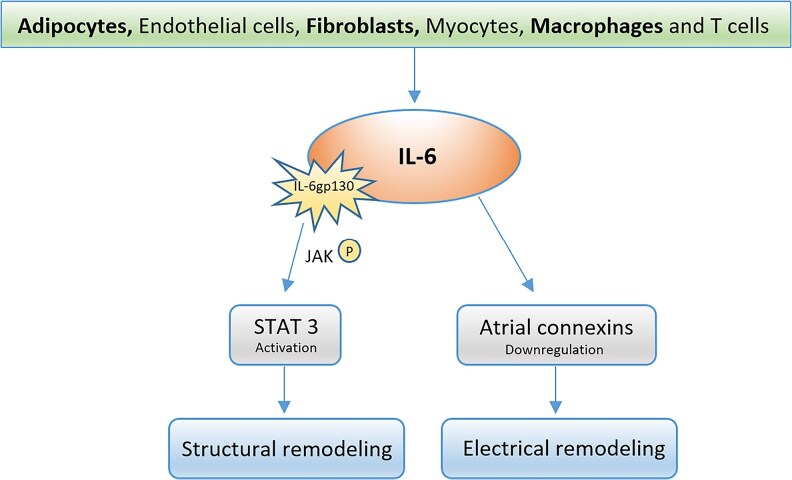
Pathophysiological involvement of IL-6 in cardiac remodelling. (The signal transducer and activator of transcription 3 (STAT3) is activated through the tyrosine phosphorylation of Janus Kinase (JAK) at the IL-6-gp130 receptor system, leading to a cascade of events that promotes the transcription of cardiac remodelling associated genes.^21^ IL-6 is directly involved not only in the structural but also in the electrical remodelling by down-regulation of atrial connexins, an anatomical component of the hemichannels forming the gap-junction mediating electrical coupling between adjacent cardiomyocytes.^22^)

IL-6 plays a key role in the regulation of inflammation and can be described as a pleiotropic cytokine. Because of its secretion by adipose tissue, it has been termed an adipokine^20^ but like other cytokines, IL-6 is secreted by many other different cells, including endothelial cells, fibroblasts, myocytes, macrophages, and T cells.^13^ IL-6 is involved in structural and electrical remodelling associated with AF^21,22^ (*Figure 4*) and it has been suggested to be an independent predictive factor for early AF recurrence after direct current cardioversion.^23^

The ability of cardiomyocytes, particularly cardiac fibroblasts, to produce IL-6 has already been demonstrated.^24^ In previous studies, higher levels of inflammatory markers, including IL-6, have been shown in blood samples from patients during AF in comparison to those with sinus rhythm.^25^ In line with this, IL-6 in our study appeared in a higher concentration in peripheral venous samples from patients with PeAF than PAF, which could be explained by the previously suggested mechanism by which AF promotes inflammation. The fact that IL-6 can be produced and secreted by a large number of cells in the body^13,20^ could explain why higher levels of IL-6 were found in peripheral blood samples obtained from the FV in comparison to the blood samples obtained from the CS. Another indicator of this non-direct pattern of IL-6 in AF is the heterogeneity shown regarding its association with AF-related stroke.^11^

#### Interleukin 18

Produced from macrophages and other cell types,^26^ IL-18 is a cytokine that has been shown to promote AF.^27^ Patients with AF present higher levels of circulating IL-18 compared with those in sinus rhythm. When comparing IL-18 levels in patients with PAF and patients with PeAF, a higher concentration in PeAF patients has been reported.^26^ Consistent with that, our study shows a significantly higher concentration in PeAF patients compared with PAF patients independent of the sampled site.

Higher levels of IL-18 have been positively related to the diameter of the left atrium in previous studies.^28^ Liu *et al*.^27^ showed a higher concentration of IL-18 in the left atrium than in peripheral venous blood. Although adipokines and other inflammatory cytokines, including IL-18, may be produced and secreted at the local level in the atrium, as well as by the surrounding epicardial adipose tissue,^27^ we did not find a significant difference in concentration between blood samples taken from the CS and FV in the overall population in our study. Nevertheless, IL-18 levels in PeAF patients were significantly higher in the CS in comparison to PAF patients, as mentioned previously. The fact that almost 80% of the PeAF group were presenting AF on their arrival to the ablation lab could be a reasonable explanation for our finding.

### Fibrotic process and sympathetic stimulation, key factors in AF development and maintenance

#### Tumour necrosis factor superfamily 14

Tumour Necrosis Factor Superfamily 14 (TNFSF-14) plays an important role in the regulation of immunological and fibrotic diseases. A recent study by Wu *et al.*^29^ identified higher levels of TNFSF14 in AF patients, which were even higher in patients with PeAF compared with patients with PAF. The study indicates the ability of TNFSF-14 to contribute to the development of fibrosis in the myocardium through various suggested mechanisms involving the polarisation of M2 macrophages. This, in turn, leads to increased collagen deposition and the activation of the TGF-β/Smad pathway, which increases the levels of circulating TGF-β1, a cytokine known to be a regulator of the fibrotic process by promoting the fibroblast-to-myofibroblast transdifferentiation.^29^ The results in our study were in line with the previous findings, showing that TNFSF14 concentration was significantly higher in PeAF blood samples from both the CS and the FV compared with PAF patients. However, no significant concentration difference was present when comparing the concentration at both sites sampled in the overall population.

#### Β-nerve growth factor

β-Nerve growth factor (Β-NGF) is a polypeptide directly involved in neuronal growth, differentiation, and survival. It is expressed in the normal heart, exhibiting higher concentration under physiological circumstances in the heart than in peripheral blood. Β-NGF has a pro-fibrogenic effect, not only in the lungs and the skin but also in the cardiac fibroblasts, which constitute the majority of the cell population in the heart. Cardiac fibroblasts can be both origins and recipients of cardiac fibrosis in the course of pathological remodelling of the myocardium.^30^

Previous experiments in mice have proven that β-NGF overexpression in the heart was associated with sympathetic hyperinnervation as well as with hypertrophy and fibrosis of the right atrium and right ventricle.^31^ Plasma levels of β-NGF have furthermore been investigated in patients after catheter ablation, where β-NGF showed a strong association with high sympathetic nerve activity.^32^ Several studies have indicated that myocardial injuries, whether ischaemic or mechanical during ablation, result in the sprouting of cardiac nerves and sympathetic hyperinnervation in animals. This phenomenon has also been associated with an elevation of β-NGF.^33^

In this study, we found a higher concentration of β-NGF in blood samples taken from the CS in patients with PeAF than in PAF but no significant concentration difference between the two sampled sites. These results may suggest that PeAF is associated with a higher level of sympathetic innervation and cardiac fibrosis compared with PAF.

#### Fibroblast growth factor 23

Fibroblast Growth Factor 23 (FGF-23) is involved in the regulation of cell proliferation and phosphorous homeostasis. Higher levels of FGF-23, common in patients with chronic kidney disease (CKD), are associated with a higher incidence of HF and AF, but the exact mechanism is poorly understood.^34^ It has been proposed that FGF-23 might induce left ventricular hypertrophy (LVH) by activating fibroblast growth factor receptor 4 on cardiac myocytes,^35^ and that FGF-23 can trigger LVH and a decline in left atrial emptying fraction, irrespective of the left ventricular structure and function in mice.^34^

Diverging from these results, additional studies failed to establish a direct correlation between FGF-23 levels and the occurrence of AF independent of other risk factors.^36^

Overall, FV samples showed a significantly higher concentration of FGF-23 than in CS samples, but no significant difference was found when comparing PAF and PeAF groups.

### Chemokine ligands: CCL-3, CCL-19, CCL-25, and CCL-28

Involving numerous ligands, receptors, and regulatory proteins in diverse cellular processes, the chemokine network is intricately complex. Its primary biological function is the induction of migration, particularly of leukocytes, yet its impact is greater than that. Exerting a significant physiological role, chemokines contribute to the development, homeostasis, inflammation, tissue repair and several processes of the innate and adaptive immunity.^37^ In this study, CCL-3 showed a higher concentration in both sampled sites from PeAF patients and no significant concentration difference when comparing the sampled locations. Some other chemokine ligands did not stand out when comparing PAF and PeAF groups, but the CS: FV ratio did show a significant concentration difference. CCL-19 showed a significantly higher presence in peripheral samples, while the concentration of CCL-25 and CCL-28 was significantly higher in the CS, which suggests a local production of these cytokines at the level of the atria. Further investigations on the specific interactions between members of the chemokine network might elucidate a relationship between these inflammatory proteins and the development of AF.

## Investigational significance

The findings obtained after analyzing this wide range of inflammatory biomarkers not only support some of the previous evidence in the field but also identify new pathways that may be involved in the development and progression of AF, representing a step forward in the research-oriented towards both the prevention of AF and the progression from PAF to PeAF.

Ideally, these findings should be validated and may eventually allow the utilisation of some of these proteins for predictive or prognostic use to potentially assess the probability of AF progression from PAF to PeAF. The use of biomarkers in risk scores in AF has shown promising results in other settings.^38^ Such a concept may also provide decision support regarding rhythm control strategies in patients with AF based on the number of risk factors, elevated inflammatory biomarkers, and low or moderate grade of symptoms.

## Limitations

This study has potential limitations, as it is a single-centre observational cohort study with a moderate sample size and single time-point measurements.

A significantly higher proportion of the PeAF patients presented with AF to the ablation lab, which according to the hypothesis supporting the contribution of AF to inflammation^14,15,16^ should be considered as a potential limitation.

While the comparison between PAF and PeAF provides valuable insights into disease progression, the inclusion of a control group without AF would have allowed for a more comprehensive assessment of whether the observed biomarker changes are specific to AF or part of a broader systemic inflammatory response. Although the aim of the study is purely descriptive, the lack of eGFR-adjusted regression modelling and control for additional confounders represent a limitation to the study.

Compared with PAF patients, PeAF patients had a higher incidence of comorbidities and therefore medications, including heart failure therapy. Evidence supports that angiotensin II promotes leucocyte migration and activation, as well as tissue infiltration, contributing to cardiac remodelling, by increasing the expression of specific chemokines.^39^ The anti-inflammatory effect of angiotensin-converting enzyme inhibitors and angiotensin receptor blocker have been shown to reduce the pro-inflammatory effect of angiotensin II in vascular endothelium and prevent cardiac remodelling.^40^ Recent studies have also demonstrated a significant impact of sodium-glucose cotransporter-2 in the reduction of pro-inflammatory cytokines.^41,42^

Although both PeAF and PAF have a similar percentage of patients medicated with statins, their anti-inflammatory effect by decreasing cytokines such as IL-6, IL-8 and monocyte chemoattractant protein −1^43^ should be taken into consideration as a possible limitation. Medication with other anti-inflammatory drugs, such as cortisone and methotrexate, was used by <5% of the patients. While this small proportion suggests a negligible impact, potential interference with the results cannot be entirely ruled out and therefore should be considered as a potential limitation.

The comorbidities taken into consideration in the study were those that represent a risk factor for cardiovascular diseases. However, the analysis did not consider other diseases that may be strongly associated with chronic inflammation.

The identification of novel inflammatory proteins that may be associated with AF pathogenesis, such as CCL3, CCL19, CCL25, CCL28, and ARTN, should be carefully interpreted, considering that no previous studies have described these associations and that chemokines are widely involved in most inflammatory processes, therefore possibly exhibiting a low grade of specificity towards AF.

## Conclusions

Evidence supports that atrial structural alterations in AF patients are induced by pro-fibrotic inflammatory proteins. Our study supports the notion of systemic association of TNFSF-14, IL-6, and IL-18 in the pathogenesis and maintenance of PeAF. Furthermore, it suggests that β-NGF and TGF-α may be associated with the pathogenesis and maintenance of PeAF at a local level in the heart, as they are expressed at a higher concentration in the CS in PeAF patients as compared with patients with PAF.

## Supplementary Material

oeaf089_Supplementary_Data

## Data Availability

The data supporting the findings of this study are available upon reasonable request.

## Patient and public involvement

Patients will be informed about the findings of this study as they were not involved in the design of it.
