# Supplementary figures and images for: Differences in inflammation biomarkers between patients with paroxysmal and persistent atrial fibrillation in the femoral vein and coronary sinus blood samples; a cohort study
Source: Eur Heart J Open. 2025 Jul 15;5(5):oeaf089. doi: 10.1093/ehjopen/oeaf089 (PMC12448471; doi:10.1093/ehjopen/oeaf089)

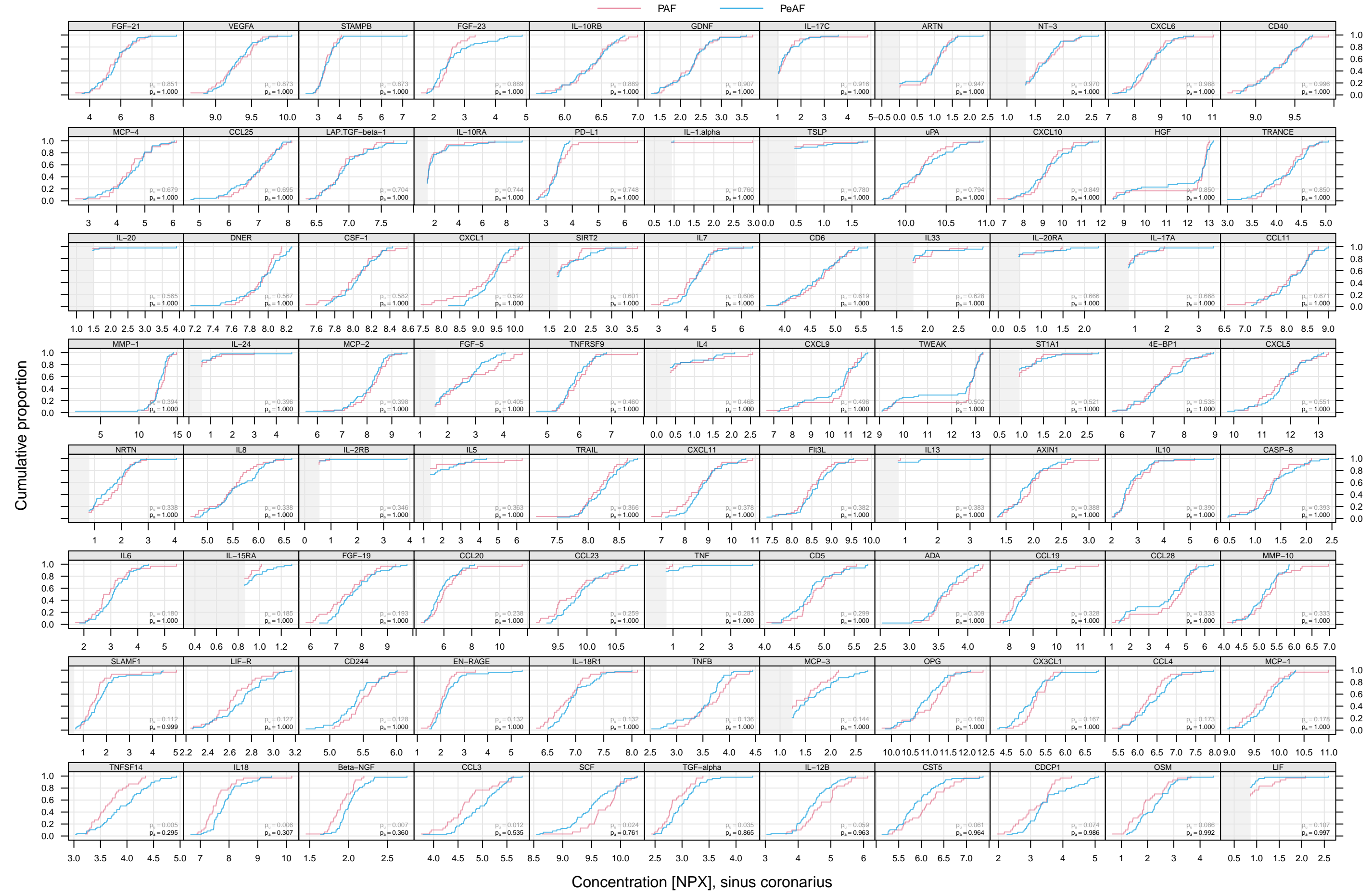

Supplement: oeaf089_Supplementary_Data [file oeaf089_supplementary_data.zip › Supplement 1.pdf]

PAF PeAF

Cumulative proportion

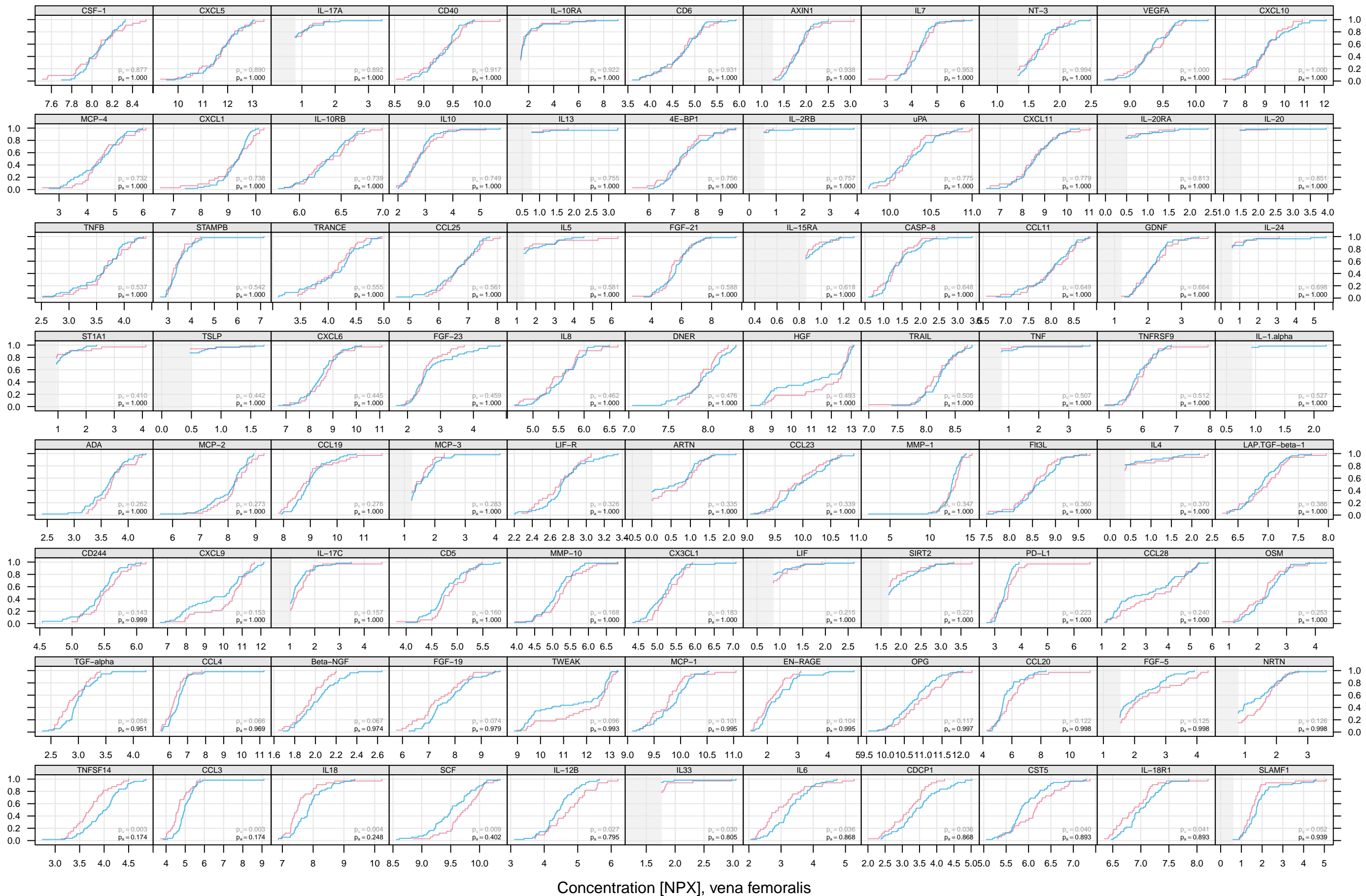

Supplement: oeaf089_Supplementary_Data [file oeaf089_supplementary_data.zip › Supplement 2.pdf]
