# Supplementary material for: Differences in inflammation biomarkers between patients with paroxysmal and persistent atrial fibrillation in the femoral vein and coronary sinus blood samples; a cohort study
Source: Eur Heart J Open. 2025 Jul 15;5(5):oeaf089. doi: 10.1093/ehjopen/oeaf089 (PMC12448471; doi:10.1093/ehjopen/oeaf089)
Supplement: oeaf089_Supplementary_Data [file Supplement 3.pdf › Supplement 3.pdf]

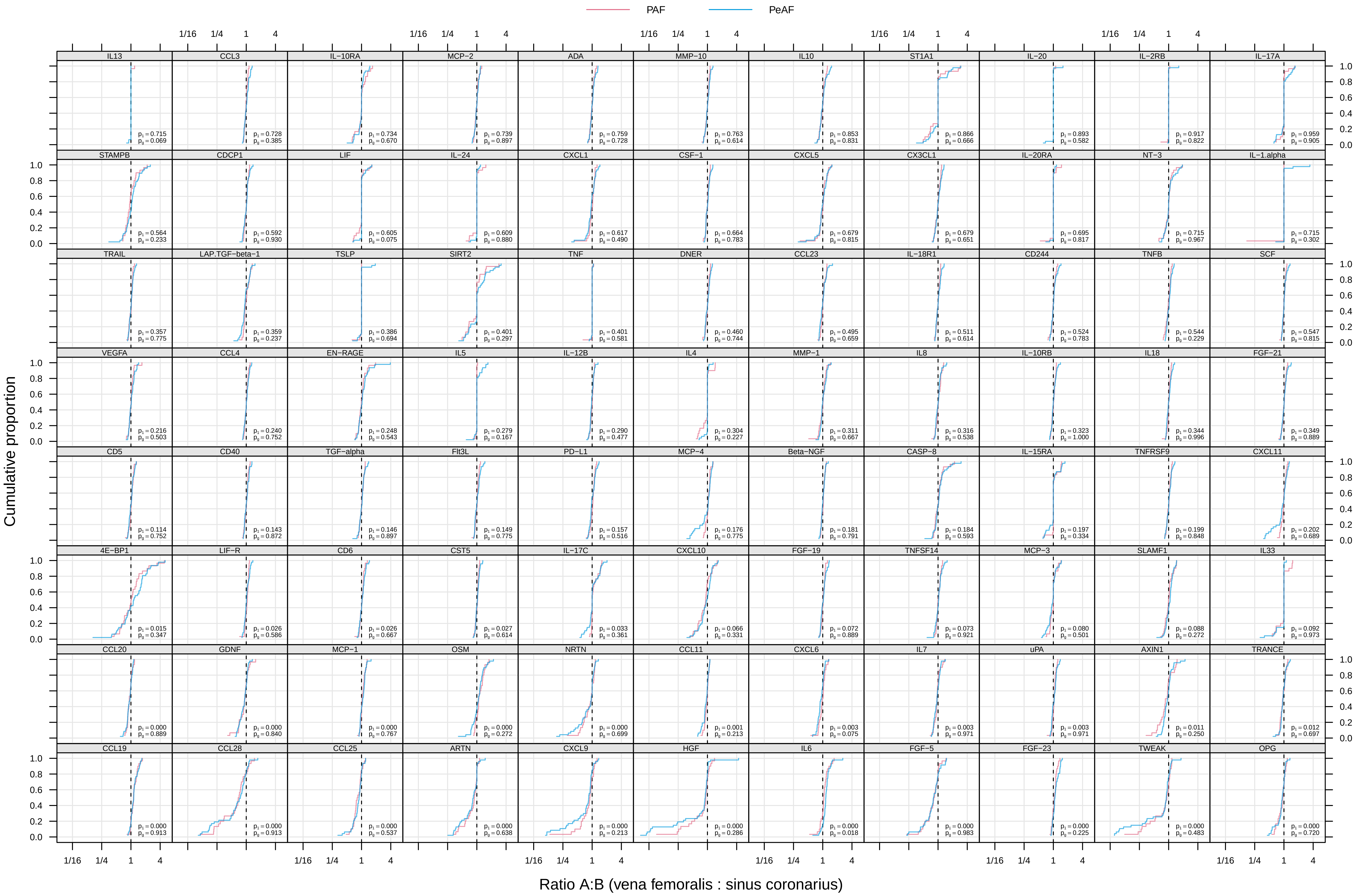

PAF
PeAF
Cumulative proportion
Ratio A:B (vena femoralis : sinus coronarius)
1/16 1/4 1 4
0.0 0.2 0.4 0.6 0.8 1.0
IL13 $p_1 = 0.715$ $p_g = 0.069$
CCL3 $p_1 = 0.728$ $p_g = 0.385$
IL−10RA $p_1 = 0.734$ $p_g = 0.670$
MCP−2 $p_1 = 0.739$ $p_g = 0.897$
ADA $p_1 = 0.759$ $p_g = 0.728$
MMP−10 $p_1 = 0.763$ $p_g = 0.614$
IL10 $p_1 = 0.853$ $p_g = 0.831$
ST1A1 $p_1 = 0.866$ $p_g = 0.666$
IL−20 $p_1 = 0.893$ $p_g = 0.582$
IL−2RB $p_1 = 0.917$ $p_g = 0.822$
IL−17A $p_1 = 0.959$ $p_g = 0.905$
STAMPB $p_1 = 0.564$ $p_g = 0.233$
CDCP1 $p_1 = 0.592$ $p_g = 0.930$
LIF $p_1 = 0.605$ $p_g = 0.075$
IL−24 $p_1 = 0.609$ $p_g = 0.880$
CXCL1 $p_1 = 0.617$ $p_g = 0.490$
CSF−1 $p_1 = 0.664$ $p_g = 0.783$
CXCL5 $p_1 = 0.679$ $p_g = 0.815$
CX3CL1 $p_1 = 0.679$ $p_g = 0.651$
IL−20RA $p_1 = 0.695$ $p_g = 0.817$
NT−3 $p_1 = 0.715$ $p_g = 0.967$
IL−1.alpha $p_1 = 0.715$ $p_g = 0.302$
TRAIL $p_1 = 0.357$ $p_g = 0.775$
LAP.TGF−beta−1 $p_1 = 0.359$ $p_g = 0.237$
TSLP $p_1 = 0.386$ $p_g = 0.694$
SIRT2 $p_1 = 0.401$ $p_g = 0.297$
TNF $p_1 = 0.401$ $p_g = 0.581$
DNER $p_1 = 0.460$ $p_g = 0.744$
CCL23 $p_1 = 0.495$ $p_g = 0.659$
IL−18R1 $p_1 = 0.511$ $p_g = 0.614$
CD244 $p_1 = 0.524$ $p_g = 0.783$
TNFB $p_1 = 0.544$ $p_g = 0.229$
SCF $p_1 = 0.547$ $p_g = 0.815$
VEGFA $p_1 = 0.216$ $p_g = 0.503$
CCL4 $p_1 = 0.240$ $p_g = 0.752$
EN−RAGE $p_1 = 0.248$ $p_g = 0.543$
IL5 $p_1 = 0.279$ $p_g = 0.167$
IL−12B $p_1 = 0.290$ $p_g = 0.477$
IL4 $p_1 = 0.304$ $p_g = 0.227$
MMP−1 $p_1 = 0.311$ $p_g = 0.667$
IL8 $p_1 = 0.316$ $p_g = 0.538$
IL−10RB $p_1 = 0.323$ $p_g = 1.000$
IL18 $p_1 = 0.344$ $p_g = 0.996$
FGF−21 $p_1 = 0.349$ $p_g = 0.889$
CD5 $p_1 = 0.114$ $p_g = 0.752$
CD40 $p_1 = 0.143$ $p_g = 0.872$
TGF−alpha $p_1 = 0.146$ $p_g = 0.897$
Flt3L $p_1 = 0.149$ $p_g = 0.775$
PD−L1 $p_1 = 0.157$ $p_g = 0.516$
MCP−4 $p_1 = 0.176$ $p_g = 0.775$
Beta−NGF $p_1 = 0.181$ $p_g = 0.791$
CASP−8 $p_1 = 0.184$ $p_g = 0.593$
IL−15RA $p_1 = 0.197$ $p_g = 0.334$
TNFRSF9 $p_1 = 0.199$ $p_g = 0.848$
CXCL11 $p_1 = 0.202$ $p_g = 0.689$
4E−BP1 $p_1 = 0.015$ $p_g = 0.347$
LIF−R $p_1 = 0.026$ $p_g = 0.586$
CD6 $p_1 = 0.026$ $p_g = 0.667$
CST5 $p_1 = 0.027$ $p_g = 0.614$
IL−17C $p_1 = 0.033$ $p_g = 0.361$
CXCL10 $p_1 = 0.066$ $p_g = 0.331$
FGF−19 $p_1 = 0.072$ $p_g = 0.889$
TNFSF14 $p_1 = 0.073$ $p_g = 0.921$
MCP−3 $p_1 = 0.080$ $p_g = 0.501$
SLAMF1 $p_1 = 0.088$ $p_g = 0.272$
IL33 $p_1 = 0.092$ $p_g = 0.973$
CCL20 $p_1 = 0.000$ $p_g = 0.889$
GDNF $p_1 = 0.000$ $p_g = 0.840$
MCP−1 $p_1 = 0.000$ $p_g = 0.767$
OSM $p_1 = 0.000$ $p_g = 0.272$
NRTN $p_1 = 0.000$ $p_g = 0.699$
CCL11 $p_1 = 0.001$ $p_g = 0.213$
CXCL6 $p_1 = 0.003$ $p_g = 0.075$
IL7 $p_1 = 0.003$ $p_g = 0.971$
uPA $p_1 = 0.003$ $p_g = 0.971$
AXIN1 $p_1 = 0.011$ $p_g = 0.250$
TRANCE $p_1 = 0.012$ $p_g = 0.697$
CCL19 $p_1 = 0.000$ $p_g = 0.913$
CCL28 $p_1 = 0.000$ $p_g = 0.913$
CCL25 $p_1 = 0.000$ $p_g = 0.537$
ARTN $p_1 = 0.000$ $p_g = 0.638$
CXCL9 $p_1 = 0.000$ $p_g = 0.213$
HGF $p_1 = 0.000$ $p_g = 0.286$
IL6 $p_1 = 0.000$ $p_g = 0.018$
FGF−5 $p_1 = 0.000$ $p_g = 0.983$
FGF−23 $p_1 = 0.000$ $p_g = 0.225$
TWEAK $p_1 = 0.000$ $p_g = 0.483$
OPG $p_1 = 0.000$ $p_g = 0.720$
